# Post-warming quality of goat oocytes under heat shock stress: A study of the maturation rate, heat shock protein-70, adenosine triphosphate, and glutathione levels

**DOI:** 10.14202/vetworld.2025.2127-2135

**Published:** 2025-07-30

**Authors:** Widjiati Widjiati, Ninik Darsini, Viski Fitri Hendrawan, Sultan Fadhilla Taqwa, Zahra Shabira, Devia Yoanita Kurniawati

**Affiliations:** 1 Department of Veterinary Anatomy, Faculty of Veterinary Medicine, Universitas Airlangga, Surabaya, Indonesia; 2 Department of Medical Biology, Faculty of Medicine, Universitas Airlangga, Surabaya, Indonesia; 3 Department of Veterinary Reproduction, Faculty of Veterinary Medicine, Universitas Brawijaya, Malang, Indonesia

**Keywords:** adenosine triphosphate, cryopreservation, cryoprotectant, genetic conservation, glutathione, heat shock, heat shock protein 70, Kacang goat, One Health, oocyte maturation

## Abstract

**Background and Aim:**

Indonesia’s indigenous Kacang goat population is in decline, posing a threat to food security and genetic diversity. *In vitro* maturation and cryopreservation techniques are key strategies for genetic conservation. However, heat shock stress during cryopreservation can compromise oocyte viability. This study evaluates the post-warming quality of Kacang goat oocytes exposed to different cryoprotectants. This study aimed to compare the efficacy of a modified cryoprotectant (30% ethylene glycol + 1M sucrose) with a commercial cryoprotectant in preserving post-warming oocyte quality, based on maturation rates and biomarker levels (heat shock protein 70 [HSP70], adenosine triphosphate [ATP], and glutathione [GSH]).

**Materials and Methods:**

Oocytes were collected from goat ovaries and matured *in vitro* for 22 h. They were divided into three groups: Control (no vitrification), commercial cryoprotectant (T1), and modified cryoprotectant (T2). Post-warming quality was assessed using an enzyme-linked immunosorbent assay to quantify HSP70, ATP, and GSH levels. Statistical analysis included one-way analysis of variance and Pearson’s correlation (p < 0.05).

**Results:**

Maturation rates were comparable across groups (control group [CG]: 84.3%, T1: 79.8%, T2: 77.2%; p > 0.05). HSP70 levels were significantly elevated in T2 compared to CG (p < 0.05). T2 also showed significantly higher ATP (52.13 ± 7.7 ng/mL) and GSH (1.27 ± 0.66 ng/mL) levels compared to T1 (ATP: 25.65 ± 1.63; GSH: 0.06 ± 0.01 ng/mL; p < 0.05). A positive correlation was found between ATP and GSH (p = 0.014).

**Conclusion:**

The modified cryoprotectant formulation offered superior protection against cryo-induced stress, maintaining higher ATP, GSH, and HSP70 levels post-warming. This formulation holds promise for improving oocyte cryopreservation protocols and conserving the genetic resources of the Kacang goat. Further studies should assess long-term developmental outcomes.

## INTRODUCTION

Goats are a vital component of rural livelihoods in Indonesia, contributing significantly to household income and national food security. However, the sustainability of local goat farming is under threat, evidenced by a 35% decline in the native goat population between 2018 and 2023. This reduction is partly attributed to the growing preference among farmers for imported goat breeds that offer higher meat yields [1]. While the Kacang goat remains the predominant local meat-producing variety in Indonesia, it is also among the most endangered indigenous breeds due to the declining population of its purebred lineage. Recognized by its tricolor coat (black, white, and brown), slightly swollen ears, and small body size averaging 24.63 kg, the Kacang goat is a distinct and valuable genetic resource [2]. Although still commonly raised in agricultural regions such as East Java – including Surabaya – its genetic purity is increasingly compromised due to widespread crossbreeding with high-yielding breeds such as Etawa, Senduro, and Boer [1–3].

To counteract this genetic erosion, *in vitro* maturation (IVM) has emerged as a promising strategy for preserving the Kacang goat’s genetic integrity. IVM entails harvesting oocytes from female goats, maturing them *in vitro*, and either fertilizing them with sperm from purebred males or preserving them through cryopreservation for future breeding [4]. Vitrification, a form of cryopreservation using liquid nitrogen and specialized cryoprotectants, is employed to protect oocytes from cellular damage during freezing [5]. Cryoprotectants such as dimethyl sulfoxide (DMSO), ethylene glycol (EG), propanediol, and glycerin function by preventing thermal shock at −196°C [6]. However, their effectiveness varies depending on species-specific oocyte sensitivity to cryoprotectant composition and temperature shifts during the freeze-thaw cycle [7].

EG is preferred for goat oocytes due to its rapid penetration of cell membranes, which minimizes intracellular ice formation. Nevertheless, its fast transport across membranes can induce osmotic stress. Combining EG with sucrose, a non-permeable cryoprotectant, helps increase extracellular osmolality and stabilizes the surrounding environment, enhancing vitrification outcomes [7, 8]. Notably, a cryoprotectant mixture of 30% EG and 1M sucrose has been shown to reduce the expression of apoptosis-inducing factor in Kacang goat oocytes, suggesting a protective effect during cryopreservation [9].

In evaluating oocyte viability and stress response post-warming, three biomarkers are particularly important: Heat shock protein 70 (HSP70), adenosine triphosphate (ATP), and glutathione (GSH). HSP70 serves as a molecular chaperone, mitigating oxidative damage and enhancing the function of cellular antioxidant systems, including GSH. ATP, in turn, supplies the energy required for HSP70 activity and cellular repair mechanisms. As reported by Zhang *et al*. [10], the coordinated action of HSP70, ATP, and GSH is critical in protecting oocytes from oxidative stress. ATP powers HSP70’s refolding of misfolded proteins, while GSH provides antioxidant protection in synergy with HSP70, collectively enhancing cellular defense mechanisms [11, 12].

Despite ongoing efforts to conserve the genetic diversity of Indonesia’s indigenous Kacang goat through reproductive biotechnology, significant challenges remain in optimizing cryopreservation techniques – particularly vitrification protocols tailored to species-specific cellular responses. While vitrification is widely applied in oocyte preservation, much of the research and commercial formulations are developed based on human or bovine models, which may not translate effectively to goat oocytes, especially those of indigenous breeds with unique physiological characteristics. Studies on the efficacy of commonly used cryoprotectants in Kacang goat oocytes are limited, particularly concerning their influence on post-warming cellular quality markers such as HSP70, ATP, and GSH. Furthermore, although previous work has identified the role of HSP70 in cellular stress response and apoptosis regulation, few studies have examined the integrated biomolecular interaction between HSP70, ATP, and GSH in the context of vitrification stress in goat oocytes. There is also a lack of comparative analysis between commercial cryoprotectant solutions and modified formulations – such as the combination of 30% EG and 1M sucrose – specifically designed to reduce osmotic stress and oxidative damage. This gap in empirical data limits the development of optimized cryopreservation protocols needed for the effective conservation of the Kacang goat’s genetic material.

The present study aims to evaluate the efficacy of a modified cryoprotectant formulation – comprising 30% EG and 1M sucrose – in preserving the quality of Kacang goat oocytes following vitrification and warming. Specifically, the study seeks to compare this formulation to a commercially available cryoprotectant by assessing post-warming maturation rates and quantifying key biomarkers associated with oxidative stress response and cellular viability: HSP70, ATP, and GSH. By analyzing the interrelationship between these biomarkers, the study also aims to elucidate the potential mechanistic pathways through which cryoprotectants influence oocyte quality. This research not only contributes to the refinement of species-specific vitrification protocols but also provides critical insight into the molecular responses of indigenous goat oocytes under cryo-induced stress. The findings are expected to support more effective conservation strategies for the Kacang goat and enhance the applicability of assisted reproductive technologies in small ruminants.

## MATERIALS AND METHODS

### Ethical approval

This study received ethical clearance from the Animal Care and Utilization Committee of the Faculty of Veterinary Medicine, Universitas Airlangga (Approval No. 1. KEH. 051. 03.2023).

### Study period and location

The research was conducted from June 2023 to March 2024 at Pegirian Slaughterhouse and the Faculty of Veterinary Medicine, Universitas Airlangga, including the *in vitro* Laboratory and the Animal Pathology Laboratory.

### Ovary collection and oocyte retrieval

Goat ovaries were collected in flasks containing 0.9% NaCl solution supplemented with antibiotics and maintained at 37°C during transport to the *in vitro* laboratory. Upon arrival, the ovaries were rinsed with sterile phosphate-buffered saline (PBS), and follicles measuring 2–6 mm in diameter were aspirated using an 18G needle attached to a 10 mL syringe pre-filled with 1 mL of sterile PBS. Oocytes with 2–3 layers of cumulus cells and homogenous, intact cytoplasm were selected for further processing. All personnel involved were trained in standardized procedures for oocyte handling, cryopreservation, and biomarker analysis.

### IVM of oocytes

A total of 100 ovaries were used. From 20 ovaries, approximately 80–100 viable oocytes were typically retrieved. Selected oocytes were randomly allocated to three experimental groups. IVM was conducted in 65-mm Petri dishes containing Universal IVF Medium overlaid with mineral oil (FertiPro N.V., Beernem, Belgium). The oocytes were incubated for 22 h at 38.5°C in a humidified atmosphere containing 5% CO_2_.

### Maturation assessment

Post-incubation, cumulus cells were removed using a 30-s enzymatic denudation with HYASE-10x (Vitrolife, Gothenburg, Sweden), and the oocytes were transferred to a fresh medium. Maturation was evaluated by the presence of the first polar body (PB1), indicating progression to the metaphase II stage, using an Olympus X41 inverted microscope (Evident Scientific, Tokyo, Japan).

### Experimental grouping

- Group 1 (control group [CG]): No vitrification
- Group 2 (T1): Vitrified with a commercial cryoprotectant
- Group 3 (T2): Vitrified with a modified cryoprotectant (30% EG + 1M sucrose).

### Vitrification and warming protocols

#### Treatment group 1 (T1): Commercial cryoprotectant

Oocytes were equilibrated in the commercial solution for 12–15 min and then exposed to the vitrification solution for 1 min. They were loaded into hemistraws within 0.5-mm straws and stored in liquid nitrogen at −196°C for 7 days. During warming, oocytes were sequentially exposed to thawing (1 min), dilution (3 min), warming solution 1 (5 min), and warming solution 2 (1 min) to ensure gradual rehydration and minimize osmotic shock.

#### Treatment group 2 (T2): Modified cryoprotectant (30% EG + 1M sucrose)

Following the protocol in Patent IDP00008642 (Directorate General of Intellectual Property, Ministry of Law and Human Rights of the Republic of Indonesia), oocytes were exposed to 30% EG for 15–18 min and then to 1M sucrose for 30 s. They were loaded into hemistraws and vitrified similarly to T1. The warming protocol involved sequential immersion in 0.25M, 0.5M, and 1M sucrose solutions for 1, 2, and 3 min, respectively, to ensure controlled rehydration [13, 14].

### Sample preparation for enzyme-linked immunosorbent assay (ELISA)

Post-warming, 80 morphologically normal oocytes were selected and divided into eight Eppendorf tubes (Eppendorf, Hamburg, Germany) (10 oocytes/tube). Lysis buffer was added, and samples were vortexed for 15–20 min. Following centrifugation at 1,398 × *g* for 10 min, the supernatants were collected for biomarker quantification.

### Biomarker quantification: ELISA for HSP70, ATP, and GSH

ELISA assays were conducted to quantify:

- HSP70: Rat HSP70/HSPAS9 ELISA kit (E-EL-R0479, Elabscience)
- ATP: Rat ATP ELISA kit (E0665Mo, BT Lab)
- GSH: GSH ELISA kit (E-EL-0026, BT Lab).

For each biomarker, 50 μL of standards or samples was added in duplicate to 16 wells. After adding biotinylated antibody, plates were incubated at 37°C for 60 min, followed by five washes. Streptavidin-horseradish peroxidase (Thermo Fisher Scientific, Waltham, MA, USA) was added and incubated, followed by the addition of substrate solutions A and B. The reaction was stopped after color development, and absorbance was read at 450 nm. Sample concentrations were derived using standard curves. Outliers were excluded based on standard curve deviation.

### Experimental workflow overview

**Figure 1:**
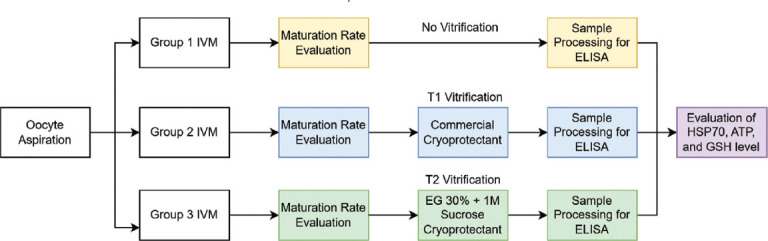
Experimental design for evaluating oocyte maturation rates and oxidative stress biomarkers. This section outlines the division of oocytes into three groups for *in vitro* maturation, subsequent maturation rate evaluation, cryoprotectant exposure (for Groups 2 and 3), sample processing for ELISA, and final evaluation of HSP70, ATP, and GSH levels. ELISA=Enzyme-linked immunosorbent assay, HSP70=Heat shock protein 70, ATP=Adenosine triphosphate, GSH=Glutathione.

As shown in Figure 1, the workflow involved:

1. IVM of oocytes (Groups 1–3)
2. Vitrification/warming protocols (T1 and T2)
3. Assessment of maturation rates and selection of oocytes
4. ELISA analysis of HSP70, ATP, and GSH as markers of oxidative stress and oocyte quality.

### Statistical analysis

Normality and homogeneity were tested using the Shapiro–Wilk and Levene’s tests, respectively. Since the data were normally distributed and homogenous (p > 0.05), one-way analysis of variance was applied. Significance was set at p < 0.05. Pearson’s Chi-square test was used for correlation analysis between biomarkers. Analyses were performed using IBM SPSS version 2023 (IBM Corp., NY, USA).

## RESULTS

### Study design and objectives

This study evaluated the effects of heat shock stress on Kacang goat oocyte quality by quantifying three key biomarkers: HSP70, ATP, and GSH. The primary objective was to compare the efficacy of two cryoprotectants – a commercial formulation and a modified combination of 30% EG and 1 M sucrose – in preserving oocyte quality post-warming.

### Oocyte maturation rates

The number of oocytes collected, matured, and the maturation rates across the three experimental groups are summarized in Table 1. In Group 1 (CG), 140 oocytes were collected, of which 118 matured, yielding a maturation rate of 84.3%. In Group 2 (treatment group 1/T1), 163 oocytes were collected and 130 matured, resulting in a maturation rate of 79.8%. In Group 3 (treatment group 2/T2), 145 oocytes were collected and 112 matured, with a 77.2% maturation rate. The overall number of oocytes collected across all groups was 448, with 360 reaching maturation.

**Table 1:** Maturation rates of Kacang goat oocytes.

Group	Number of oocytes collected	Number of matured oocytes	Maturation rate (%)
Group 1	140	118	84.3^a^
Group 2	163	130	79.8^a^
Group 3	145	112	77.2^a^
Total	448	360	

This table presents the maturation rates of Kacang goat oocytes following a 22-h *in vitro* maturation (IVM) period before exposure to cryoprotectants. The oocytes were categorized into three groups (Group 1, Group 2, and Group 3) based on their initial IVM batch. The “Maturation Rate” is calculated as the percentage of mature oocytes relative to the total number of oocytes collected in each group. Different superscript letters within the same column indicate statistically significant differences between groups (p < 0.05). The identical superscript “a” across all maturation rates indicates that no statistically significant differences (p < 0.05) were found among the groups.

**Figure 2:**
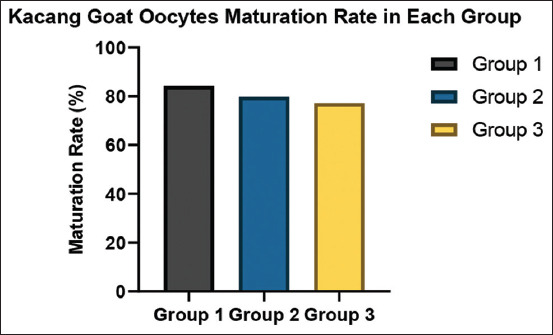
Maturation rates of Kacang goat oocytes across three different groups.

**Figure 3:**
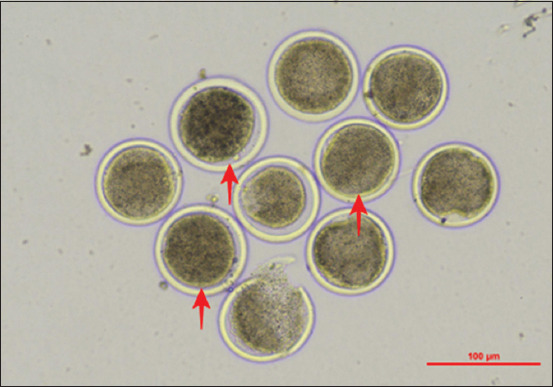
Microscopic image of mature goat oocytes after 22 h of incubation, showing the first polar body indicated by a red arrow. This result confirms successful progression to the metaphase II stage.

Statistical analysis revealed no significant differences in maturation rates between the groups (p > 0.05). These findings are illustrated in Figure 2, where Group 1 showed the highest maturation rate, followed by Groups 2 and 3. Microscopic assessment further confirmed nuclear maturation, evidenced by the extrusion of the PB1 in mature oocytes (Figure 3).

### Biomarker analysis

#### HSP70 levels

**Figure 4:**
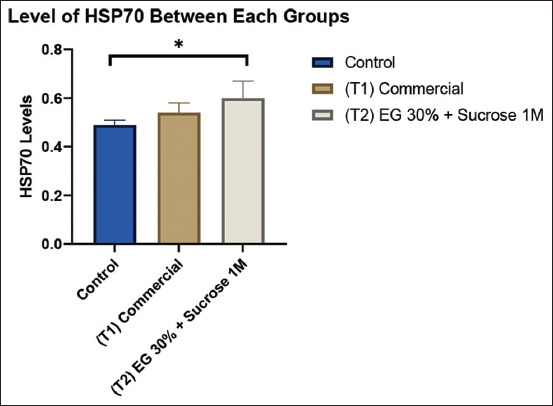
The graph compares HSP70 levels between the control group, commercial cryoprotectant (T1), and modified cryoprotectant with EG 30% and sucrose 1M (T2). The highest bar for HSP70 levels was observed in the T2 group. Signs (*) within each graph indicate significant differences (p < 0.05). HSP70=Heat shock protein 70.

As shown in Table 2 and Figure 4, HSP70 levels were significantly elevated in the T2 group (0.60 ± 0.07 ng/mL) compared to the CG (0.49 ± 0.02 ng/mL) (p < 0.05). Although the T2 group exhibited higher HSP70 levels than the T1 group (0.54 ± 0.04 ng/mL), the difference was not statistically significant (p > 0.05). Similarly, no significant difference was observed between the control and T1 groups.

**Table 2:** HSP70 levels in Kacang goat oocytes.

Group	n	HSP70 levels
Control group (CG)	8	0.49 ± 0.02^a^
Commercial (treatment 1)	8	0.54 ± 0.04^ab^
EG30% + 1M sucrose (treatment 2)	8	0.60 ± 0.07^b^

The table presents HSP70 levels difference (ng/mL, expressed as mean ± standard deviation) in Kacang goat oocytes from the Control Group (CG, non-vitrified), commercial cryoprotectant (treatment 1), and modified cryoprotectant (EG30%+1M sucrose, treatment 2) groups. The results demonstrated the highest HSP70 level in the T2 group. The different superscript letters indicate statistically significant differences (p < 0.05) among the groups. HSP70=Heat shock protein 70, EG=Ethylene glycol.

#### GSH levels

**Figure 5:**
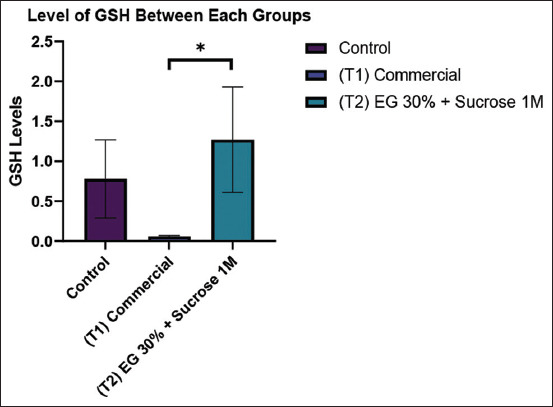
The graph compares GSH levels between the control group, commercial cryoprotectant (T1), and modified cryoprotectant with ED 30% and sucrose 1M (T2). The highest bar for GSH levels was observed in the T2 group. Signs (*) within each graph indicate significant differences (p < 0.05). GSH=Glutathione.

As presented in Table 3 and Figure 5, GSH levels were markedly higher in the T2 group (1.27 ± 0.66 ng/mL) compared to the T1 group (0.06 ± 0.01 ng/mL) (p < 0.05). However, the difference between T2 and the CG (0.78 ± 0.49 ng/mL) was not statistically significant.

**Table 3:** GSH levels in Kacang goat oocytes.

Group	n	GSH levels
Control group (CG)	8	0.78 ± 0.49^ab^
Commercial (treatment 1)	8	0.06 ± 0.01^a^
EG30% + 1M sucrose (treatment 2)	8	1.27 ± 0.66^b^

The table presents GSH levels difference (ng/mL, expressed as mean ± standard deviation) in Kacang goat oocytes from the control group (CG, non-vitrified), commercial cryoprotectant (treatment 1), and modified cryoprotectant (EG30%+1M sucrose, treatment 2) groups. The results demonstrate the highest GSH level in the T2 group. The different superscript letters indicate statistically significant differences (p < 0.05) among the groups. ATP=Adenosine triphosphate, GSH=Glutathione, EG=Ethylene glycol.

#### ATP levels

**Figure 6:**
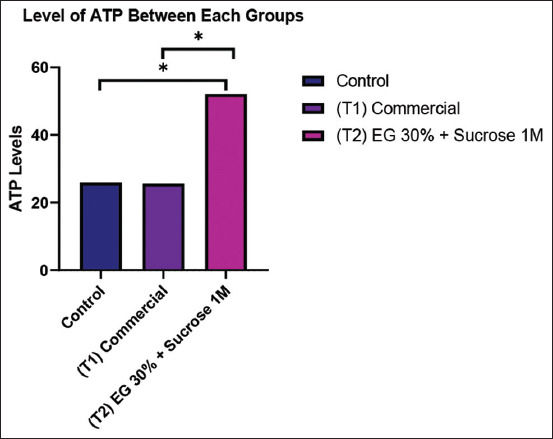
The graph compares ATP levels between the control group, commercial cryoprotectant (T1), and modified cryoprotectant with ED 30% and sucrose 1M (T2). The highest bar for ATP levels was observed in the T2 group. Signs (*) within each graph indicate significant differences (p < 0.05). ATP=Adenosine triphosphate.

According to Table 4 and Figure 6, ATP concentrations in the T2 group (52.13 ± 7.7 ng/mL) were significantly higher than those in both the CG (25.95 ± 1.95 ng/mL) and the T1 group (25.65 ± 1.63 ng/mL) (p < 0.05).

**Table 4:** ATP levels in Kacang goat oocytes.

Group	n	ATP levels
Control group (CG)	8	25.95 ± 1.95^a^
Commercial (treatment 1)	8	25.65 ± 1.63^a^
EG30% + 1M sucrose (treatment 2)	8	52.13 ± 7.7^b^

The table presents GSH levels difference (ng/mL, expressed as mean ± standard deviation) in Kacang goat oocytes from the control group (CG, non-vitrified), commercial cryoprotectant (treatment 1), and modified cryoprotectant (EG30%+1M sucrose, Treatment 2) groups. The results demonstrate the highest GSH level in the T2 group. The different superscript letters indicate statistically significant differences (p < 0.05) among the groups. ATP=Adenosine triphosphate, GSH=Glutathione, EG=Ethylene glycol.

### Correlation analysis of biomarkers

Table 5 presents the Pearson correlation analysis among the three biomarkers – HSP70, ATP, and GSH. A significant positive correlation was found between ATP and GSH levels (p = 0.014; p < 0.05), suggesting a possible relationship between cellular energy status and antioxidant capacity. However, no significant correlations were detected between HSP70 and ATP or HSP70 and GSH, indicating that HSP70 may function independently of ATP and GSH levels under the conditions tested.

**Table 5:** Correlation analysis of three biomarkers (p-value).

Biomarkers	HSP70	ATP	GSH
HSP70		0.804	0.250
ATP	0.804		0.014*
GSH	0.250	0.014*	

The table presents the p*-*values from the correlation analysis conducted between the HSP70, ATP, and GSH levels. The result shows a significant correlation between ATP and GSH. (*) indicates a statistically significant correlation between the respective biomarkers (p < 0.05). HSP70=Heat shock protein 70, ATP=Adenosine triphosphate, GSH=Glutathione.

## DISCUSSION

### Effectiveness of IVM

The maturation rate observed in this study reflects the effectiveness of the IVM process in goat oocytes [15]. Oocyte maturation involves two interconnected events – nuclear maturation, marked by germinal vesicle breakdown (GVBD) and extrusion of the PB1, and cytoplasmic maturation, regulated by calcium ion (Ca^2+^) oscillations that delay GVBD until metaphase I [16]. Synchronization of both processes is critical for successful fertilization and embryonic development. The consistent maturation rates across all groups suggest that the IVM conditions were well controlled, enabling a reliable evaluation of cryoprotectant impact on oocyte quality [17].

### Impact of cryoprotectants on cellular biomarkers

The maturation process is significantly influenced by three key molecular players – HSP70, GSH, and ATP – particularly after exposure to cryopreservation stress. ATP serves as the primary cellular energy source [18], while GSH and HSP70 protect oocytes against oxidative damage, thermal fluctuation, and metabolic stress [19]. In this study, oocytes treated with the modified cryoprotectant (30% EG + 1M sucrose, T2) showed elevated levels of all three biomarkers compared to those treated with the commercial cryoprotectant (T1), and in some cases, compared to the CG.

### Role of HSP70

HSP70 levels were significantly higher in the T2 group than in the CG, although not significantly different from T1. HSP70 plays a vital role in folliculogenesis, oogenesis, and embryo development, promoting cell survival and preventing apoptosis, especially under stress [20, 21]. It supports oocyte metabolism by preserving mitochondrial function and regulating ATP synthesis through DRP1 protein modulation [22–25]. Elevated HSP70 levels post-vitrification, particularly in the T2 group, suggest enhanced cellular protection against osmotic and thermal shock. Increased HSP70 expression is indicative of active molecular chaperoning, which refolds misfolded proteins and prevents aggregation, thereby contributing to improved post-warming oocyte quality [4, 10].

### Role of GSH

GSH levels were significantly higher in the T2 group than in T1, with no significant difference compared to the CG. GSH plays a central role in modulating cellular responses, such as proliferation, differentiation, and apoptosis [26]. As an antioxidant, it defends against ROS-induced damage to organelles and DNA [25] and maintains the integrity of the meiotic spindle, essential for chromosomal alignment [27, 28]. In synergy with HSP70, GSH undergoes glutathionylation under stress, thereby enhancing HSP70’s ATPase activity and bolstering cellular resilience [19]. Although correlation analysis did not show direct relationships between HSP70 and either GSH or ATP, this may reflect indirect or temporally offset interactions, suggesting that HSP70 might act through downstream signaling or intermediate regulatory pathways [4, 10].

### Role of ATP

ATP levels were significantly higher in the T2 group than in both CG and T1. During IVM, ATP is crucial for supporting the metabolic demands of the oocyte, including membrane transport, protein synthesis, and the maintenance of ion gradients [29, 30]. Elevated ATP in the T2 group reflects improved energy metabolism and better preservation of oocyte functionality after vitrification. Sustained ATP production indicates greater resistance to stress and enhanced cellular viability [31–33]. The absence of significant correlations between ATP and HSP70 or GSH further supports the hypothesis of multifactorial regulatory pathways.

### Comparison of cryoprotectant performance

After 22 h of IVM, oocytes underwent vitrification using either commercial or modified cryoprotectants. While cryoprotectants are essential for reducing osmotic injury during cryopreservation, they also activate cellular defense responses such as HSP70 and GSH expression. HSP70 helps maintain mitochondrial function and ATP balance, while GSH protects spindle integrity. For example, due to its high permeability, EG is considered safer than DMSO, propylene glycol (PROH), or glycerol [34]. The modified EG-sucrose formulation used in this study showed improved outcomes for oocyte viability and biomarker levels, outperforming the commercial alternative. Previous studies by Trapphoff *et al*. [35] and Zhao *et al*. [36] have reported post-vitrification survival rates over 50% in bovine and goat oocytes. Given that most commercial cryoprotectants are designed for human oocytes, their effectiveness in non-human species may be limited [37–39]. These findings support the superior protective capacity of the modified formulation for Kacang goat oocytes.

### Limitations and future directions

While this study effectively demonstrated the advantages of a modified cryoprotectant based on maturation rates and biomarker expression, several limitations remain. Notably, the study did not assess functional fertility outcomes, including fertilization success, embryo development, or live birth rates. These metrics are crucial for fully validating the developmental competence of preserved oocytes. In addition, although group sizes were standardized, the overall sample size may limit the detection of subtle differences and the broader applicability of the findings. Future studies should incorporate larger sample sizes and assess fertilization and embryo development to confirm the long-term efficacy of the modified cryoprotectant. Such research would provide deeper insight into its utility for genetic conservation and assisted reproduction in Kacang goats.

## CONCLUSION

This study demonstrated that the modified cryoprotectant formulation, containing 30% EG and 1 M sucrose, offers superior protection for Kacang goat oocytes during vitrification and warming. Compared to the commercial cryoprotectant, oocytes treated with the modified solution exhibited significantly higher levels of HSP70 (0.60 ± 0.07 ng/mL), ATP (52.13 ± 7.7 ng/mL), and GSH (1.27 ± 0.66 ng/mL), indicating enhanced resilience to heat shock and oxidative stress. Although maturation rates across all groups were not significantly different (CG: 84.3%, T1: 79.8%, T2: 77.2%), the biochemical profile of the T2 group suggests improved post-warming cellular function and viability.

The strength of this study lies in its comprehensive biomarker-based evaluation, which integrates stress response (HSP70), antioxidant capacity (GSH), and metabolic energy (ATP), providing a multidimensional understanding of oocyte quality post-vitrification. This approach allows for a more precise assessment than morphological criteria alone.

Practically, the findings highlight the potential of species-specific cryoprotectant formulations to enhance reproductive biotechnology protocols for indigenous breeds. The modified cryoprotectant may improve the success rate of oocyte preservation, *in vitro* fertilization, and genetic conservation programs, particularly for endangered or underutilized livestock, such as Kacang goat.

In conclusion, the modified EG-sucrose form-ulation offers a promising alternative to commercial cryoprotectants for preserving the viability and functional integrity of goat oocytes. Future studies should validate these findings by assessing fertilization rates, embryo development, and offspring quality to fully establish the translational potential of this cryopreservation strategy in field-level conservation and breeding programs.

## AUTHORS’ CONTRIBUTIONS

WW: Conceptualization of the study, validation, investigation, visualization, supervision of the study, methodology, formal analysis, and writing – original draft. ND and VFH: Validation, investigation, and methodology. ZS, SFT, and DYK: Visualization and drafted and revised the manuscript. All authors have read and approved the final manuscript.
